# Incidence of Hepatitis-C among HIV infected men who have sex with men (MSM) attending a sexual health service: a cohort study

**DOI:** 10.1186/1471-2334-11-39

**Published:** 2011-02-03

**Authors:** Deepa G Gamage, Tim RH Read, Catriona S Bradshaw, Jane S Hocking, Kerry Howley, Marcus Y Chen, Christopher K Fairley

**Affiliations:** 1 Melbourne Sexual Health Centre, Alfred Hospital, Melbourne, Australia; 2 Epidemiology Unit, Ministry of Health, Colombo, Sri Lanka; 3 School of Population Health, University of Melbourne, Australia; 4 Department of Epidemiology and Preventive Medicine, Monash University, Australia

## Abstract

**Background:**

We aimed to determine the incidence of Hepatitis C (HCV) infection among HIV-infected men who have sex with men (MSM) attending a Sexual Health Centre.

**Methods:**

A retrospective cohort study was carried out among HIV-infected MSM seen at least once between February 2002 and March 2010. The analysis was restricted to MSM who had had a negative HCV antibody test at least 6 months after their diagnosis for HIV. Duration of follow up was taken from the date of HIV diagnosis to the first positive or last negative HCV antibody test.

**Results:**

During the time 1445 HIV-infected men attended the clinic of whom 1065 (74%) were MSM. Of these, 869 (82%) were tested for HCV at any time after HIV diagnosis. Of these 869, 69% (620) tested HCV negative at least 6 months after their HIV diagnosis. These 620 men had a mean age of 34 years (range 17-72) at HIV diagnosis and a total of 4,359 person years (PY) of follow up. There were 40 incident cases of HCV, of which 16 were in injecting drug users (IDU) and 24 in non-IDU. The overall incidence of HCV among HIV-infected MSM was 0.9/100 PY (95% CI 0.6-1.2). The incidence among HIV-infected IDU was 4.7/100 PY (95% CI 2.7-7.5) while the incidence among HIV-infected non-IDU was 0.6/100 PY (95% CI 0.4-0.8) (hazard ratio of 8.7 and 95% CI 4.6-16.6, P < 0.001).

The majority (78%) were tested for HCV because they developed abnormal liver transaminases (n = 31) or hepatitis symptoms (n = 2), while others (n = 7) were identified through routine HCV testing.

**Conclusion:**

A considerable proportion of HIV-positive MSM who did not inject drugs contracted HCV, presumably via sexual transmission and the main trigger for investigation was abnormal liver transaminases.

## Background

Hepatitis C virus (HCV) infection is a significant health issue, particularly among individuals with HIV infection[1,2]. Co-infection with both HIV and HCV has been associated with more rapid progression to HCV-related liver disease, and increases the risk for cirrhosis and liver cancer[2,3]. Hepatitis C is a major cause of hospital admissions and is a leading cause of death among HIV-infected persons[4].

Hepatitis C infection is transmitted mainly by parenteral exposure, particularly in IDU[5]. It remains unclear whether HCV is transmitted sexually between men, and recently reviewed studies give conflicting results[6-13]. Those studies that support sexual transmission among men having sex with men (MSM) [6-10] describe multiple sex partners and other sexual practices as risks for HCV transmission. A recent study in Sydney, Australia [10] described possible sexual transmission of HCV in HIV-negative MSM who did not use injecting drugs but not among a small cohort of HIV-positive MSM.

Recently a number of bodies have recommended screening for HCV among MSM with HIV, even in the absence of any known risk factors for HCV infection[14]. Our large sample size and existing risk factor data allow us to generate tight confidence intervals around HCV transmission among MSM with HIV. We therefore carried out a retrospective cohort study to determine the incidence of possible sexual transmission among those who did not inject drugs.

## Methods

This was a retrospective cohort study of MSM with HIV infection. Individuals were eligible for the cohort if they were seen at least once at Melbourne Sexual Health Centre's (MSHC) HIV clinic between February 2002 and March 2010, and were negative for HCV antibodies at least 6 months after the date of their HIV diagnosis. This 6 month period was chosen because HCV antibodies develop in the majority of infected patients within 6 months of infection [15,16]. Individuals who tested positive for HCV antibodies at their first HCV antibody test were excluded from the cohort analysis because they could not be confirmed as incident cases. For individuals who tested negative for HCV antibodies at their last test, follow up was from the date of their HIV diagnosis to the time of their last HCV test. For individuals who tested HCV antibody positive, but who had a previous negative HCV antibody test, the follow up time was taken from the time of their HIV diagnosis to the time of their first positive HCV antibody test.

Risk factor data were extracted from the centre's computer database: Clinical Practice Management System (CPMS). Risk factor data include both MSM without IDU or MSM with IDU. Laboratory testing data for HCV antibody were extracted from the computerised records of the Victorian Infectious Diseases Laboratory (VIDRL). The medical records of incident cases of HCV were reviewed by DG and TR to determine the reasons for the HCV test and also to carefully ascertain if there was any record of IDU that may have been missed from routinely collected risk factors.

Routine annual screening for HCV began in 2008 for MSM with HIV [17]. The same laboratory (VIDRL) has been used by the clinic since 1992. Hepatitis C antibodies were analyzed by enzyme immunoassay (EIA) (Murex anti-HCV version 4.0, Abbott Diagnostics, Abbott Park, Illinois, USA) and each positive result was subjected to supplementary testing with the Bio-Rad EIA (Bio-Rad Laboratories, Hercules, California, USA). Subsequently, HCV positive cases were tested for HCV RNA using the Cobas Amplicor ^® ^version 2 polymerase chain reaction (PCR) assay (Roche Diagnostics, Branchburg, NJ, USA).

The data collected in all three sources were merged and analysed using the Statistical Package for Social Sciences (SPSS) We calculated the incidence of HCV per 100 person years and 95% confidence intervals for all MSM, MSM who reported ever IDU and MSM without a history of IDU. Hazard ratios and 95% confidence intervals were calculated to compare incidence between MSM who reported ever IDU and MSM without a history of IDU. A Kaplan-Meier curve was generated to illustrate the time to HCV acquisition for MSM who reported ever IDU and MSM without a history of IDU separately and the log rank method was used to compare these two groups. Ethics clearance was obtained from the ethics committee at the Alfred Hospital.

## Results

**Figure 1:**
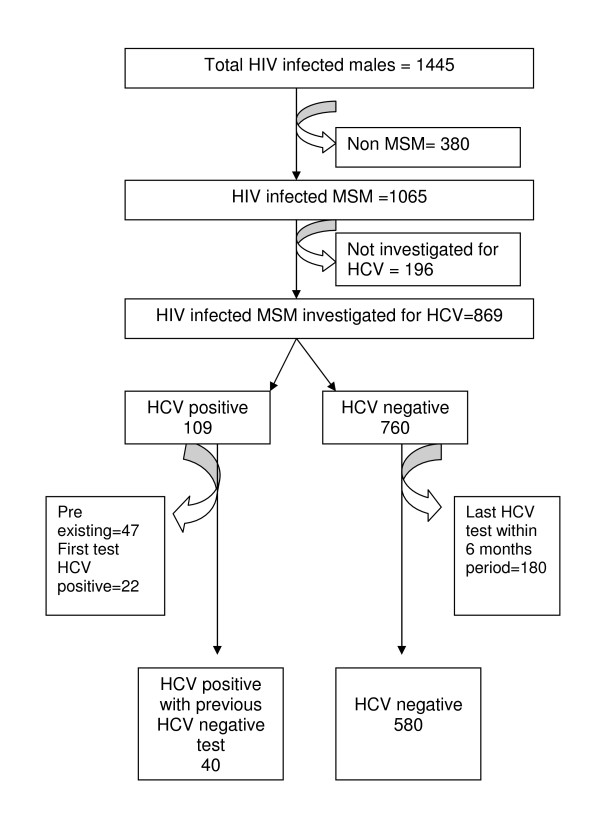
**Cohort of HIV positive MSM in identifying hepatitis C incident cases**.

There were 1445 HIV infected males seen at least once at MSHC during the eight year period. Of these 1065 (74%) were MSM and of these 869 (82%) were tested for HCV at any time after HIV diagnosis. Of these 869, we excluded 180 HCV-negatives because their last HCV test was less than 6 months after their first HIV positive test (figure 1). We also excluded 69 HCV positive cases (41 non-IDU and 28 IDU) because they had HCV at the time of or before their HIV diagnosis (n = 47) or they did not have a negative HCV test before their first positive HCV test (n = 22). Therefore 620 MSM with a HCV antibody negative test more than 6 months after their HIV diagnosis were included in this cohort.

The mean age of the 620 MSM at HIV diagnosis was 34 years (range 17-72), 434 (70%) were Australian born and 39 (6%) had ever injected drugs. The median CD4 count of the cohort on their last visit was 566 cells/mm^3 ^(inter quartile range 402 to 734 cells/mm^3^) and 342 (73%) had a HIV-VL of less than 50 copies/ml. Basic population characteristics of the cohort are given in table 1.

**Table 1:** Population characteristics of the study cohort of HIV positive MSM

Description	HCV positives (n = 40)	HCV negatives N = 580
Mean age (years) at HIV diagnosis (range)	32(20-45)	34 (17-72)

Mean age (years) at last negative HCV test (range)	39(25-64)	40(20-78)

IDU	16 (40%)	23 (4%)

Australian born	32 (80%)	402 (69%)

Most recent median CD4 cell count cells/mm^3 ^(Interquartile range)	531(358-856)	568 (404-731)

Most recent HIV viral load <50 (copies/ml)	25 (63%)	311 (54%)

There were 4359 person years (PY) of follow up during which, 40 individuals developed HCV, with an incidence of 0.9/100 PY (95% CI 0.6-1.2). Sixteen cases occurred in those who had ever IDU (341 PY of follow up) with an incidence of 4.7/100 PY (95% CI 2.7-7.5) and 24 cases occurred in those who had never IDU (4018 PY) with an incidence of 0.6/100 PY (95% CI 0.4-0.8). None of the cases had documented evidence of tattoos or blood transfusions. The incidence of HCV was significantly higher among those with a history of ever injecting drugs (HR = 8.7; 95% CI 4.6-16.6, P < 0.001). Those born in Australia compared to those born in overseas were not at greater risk of acquiring HCV (HR 1.41, 95% CI: 0.65-3.09, P = 0.32). Individuals 35 years and older were not at greater risk of HCV infection compared to those younger than 35 years (HR 1.87, 95% CI: 0.54-6.41, P = 0.63).

**Figure 2:**
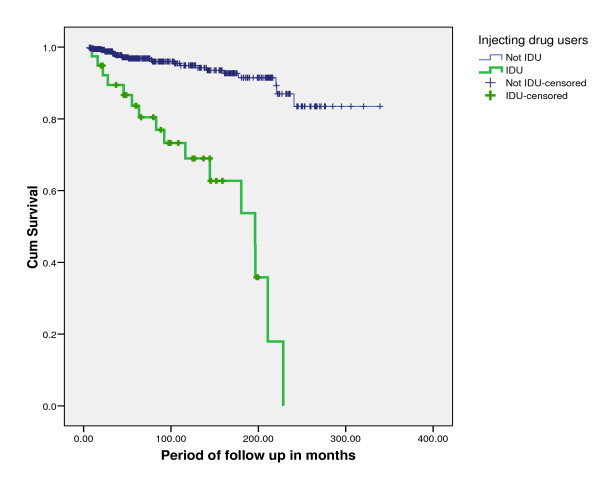
**Kaplan-Meier analysis of contraction of Hepatitis C among HIV positive MSM, comparing IDU and non-IDU**. IDU = injecting drug use. Cum Survival = Cumulative survival. Because the three IDU with the longest follow up became infected with HCV, the figure gives the impression that all IDU became infected when in fact only 16 of 39 acquired HCV infection

The Kaplan Meier curves are shown in Figure 2, and there is a significant difference in survival curves between the two groups. (P < 0.001) of IDU and non-IDU. Because the three IDU with the longest follow up became infected with HCV, the figure gives the impression that all IDU became infected when in fact only 16 of 39 acquired HCV infection.

In 25 (63%) of the 40 incident cases of HCV, a HCV-PCR test was performed and 23 (92%) were PCR positive. Medical records were reviewed for documented evidence to identify sexual health risks in transmission of HCV in 24 non-injecting HCV seroconvertors. One case reported fisting, one reported rectal bleeding and one had a penile ulcer. Two reported they had not practised fisting.

On review of the records, the reason for HCV antibody testing, in the majority of cases of HCV (83%, n = 33) was a rise in routinely tested liver enzymes (n = 31), or the development of symptoms suggesting hepatitis (n = 2). Seven (18%) asymptomatic cases were identified in routine screening for HCV and of these two had slightly elevated aminotransferase levels which were not the reason for the test. The peak median AST (aspartate aminotransferase) levels were four times the upper limit of normal (ULN) (inter quartile range (IQR) 2-8 times) and the peak median ALT (alanine aminotransferase) levels were seven times the ULN (IQR 3-18 times) among the 33 who were tested because of abnormal liver function tests or development of hepatitis symptoms.

## Discussion

Our study is one of the larger cohort studies performed to date and the first cohort study in Australia, to report presumed sexual transmission of HCV among HIV-infected MSM who do not inject drugs. Our study suggests that HIV positive MSM who have never IDU have a low but significant risk of HCV infection of about half a percent per year. Our data supports reports from other studies from the UK [6], Switzerland [7] and Amsterdam [9] which also report presumed sexual transmission in about 1 percent of HIV-infected MSM per year.

There are four published cohort studies of the incidence of HCV among HIV-infected MSM without other recognized HCV risks where it was possible to calculate an incidence per 100 person years. In the UK [6] a study of 308 HIV infected MSM with 1190 PY of follow up reported an incidence of 0.92/100PY. In Switzerland [7] a study of 1571 HIV infected MSM who reported unsafe sex (n = 414) reported an incidence of 0.7/100PY (95% CI 0.3-1.4). In Amsterdam [9] a study of 1836 MSM including 504 HIV infected MSM (between years 2000-2003) with 572 PY of follow up reported an incidence of 0.87/100PY (95% CI 0.28-2.03). An Australian study [10] of 159 HIV infected MSM with 238 PY of follow up reported no cases of HCV infection although these findings are consistent with our study because the upper limit of their 95% confidence interval was 1.54/100 PY that includes our point estimate of 0.6/100 PY.

There are three published cohort studies of the incidence of HCV among HIV negative MSM without other recognized HCV risks where it is possible to calculate an incidence per 100 person years. In the UK [18] a study of 948 HIV negative MSM who were not IDU, with 3335 PY of follow up reported an incidence of 0.15/100PY(95% CI 0.05-0.35). In Australia, a study [10] among 1383 HIV negative MSM with 4412 PY of follow up reported an incidence of 0.11/100 PY (95% CI 0.03-0.26) and only one of 5 HCV positives reported injecting drug use. In Canada a study [19] of 1053 HIV-negative MSM without other risks for HCV transmission with 2610PY of follow up reported no cases with upper limit of 95% confidence interval 0.14/100PY.

There are five published studies where it was either not possible to calculate a specific incidence of HCV among either HIV-positive or HIV-negative MSM who were not injecting drugs. In France [8], 4 cases of HCV occurred in 252 HIV-infected MSM whose follow up time was not specified although among the entire cohort of 402 HIV-positives the median follow up of 36 months. A study with 20 years of data from 12 cohorts in the CASCADE collaboration [20] 216 cases occurred among 3014 HIV-infected MSM, although data on drug injecting status was not presented. In Germany [21] a study among MSM with 10199 PY of follow up reported an incidence of between 0.36-1.05/100 PY although again the incidence according to drug injecting status was not presented. A study from Denmark [22] in the 1980s documented HCV transmission in 250 MSM without other risks but the HIV prevalence was not given. Similarly an Italian study [23] of 244 MSM without IDU documented an incidence of 1.37/100 PY, but again there was no information on HIV prevalence.

The most common reason for HCV testing among HCV positive individuals in our study was the development of abnormal liver function tests. There were seven cases identified through routine screening for HCV that only began in 2008 and it is possible that among these seven cases, infection had occurred significantly earlier. This bias is evident in the survival curve with the increase in gradient of the curve at about 200-300 months of follow up, but would have only had a minor effect on the incidence estimate. The data do however suggest that routine monitoring of liver function tests will identify the majority of cases of even asymptomatic HCV infection. This finding supports a recent study in the UK [24] where elevated liver enzymes were the most common reason for HCV diagnosis. Another study [25] among HIV positive MSM showed testing for ALT was more sensitive than HCV antibody testing for early diagnosis of HCV. However given the importance of early treatment of HCV diagnosis, clinicians should not rely only on elevations of routine liver function tests because they will miss a significant minority of cases.

Routine testing for hepatitis C among MSM began in 2008 [17] and since then, of the 444 HIV-positive HCV-negative MSM who were having bloods drawn at least once a year at our clinic, 75% (n = 332) had subsequent HCV serology. It would be helpful to put the rates of Hepatitis C infection described in our study in context with the rates of other STI in MSM in Victoria. In a study looking at the proportion of samples positive for gonorrhoea and chlamydia by anatomical site between 2002 to 2009 at our centre in MSM, no increase in either infection was noted however there was a suggestion that the proportion of samples positive for gonorrhoea may be falling (Vodstrcil LA, Fairley CK, Fehler G, Leslie D, Walker J, Bradshaw C, Hocking JS: Trends in Chlamydia and Gonorrhoea Positivity among Heterosexual Men and Men who have sex with Men attending a sexual health service between 2002 and 2009, submitted). In contrast rates of syphilis have risen significantly over this time in MSM [26,27]. The first case of anorectal LGV was detected in a MSM, Victoria in 2005 [28] and since then 18 have been diagnosed in MSM with HIV at MSHC until March 2010.

Our study had a number of weaknesses. Firstly it was retrospective and therefore excluded some individuals from the analysis who were either never tested for HCV and were HCV positive when first tested. If the incidence of HCV among those never tested was very high then we would have underestimated the true incidence. Conversely if the HCV incidence among those never tested was very low then we would have overestimated the true incidence. Our estimate would have also underestimated the true incidence if a significant proportion of those whose first HCV test was positive were true incident cases. If we included all of the 41 cases positive on their first tests it would have only increased the incidence among MSM who were non-IDU from about 0.6/100 PY to 1.7 per 100 PY. Given that the objective was to compare the incidence rates between members of the HIV positive MSM cohort who did and did not engage in IDU, we thought underestimating the incidence was preferred.

Our study did not collect detailed risk factor information prospectively on all members of the cohort and therefore this information may be incomplete, particularly in relation to the possible risk factors for sexual transmission of HCV infection. We are therefore unable to look at hazard ratios for different sexual exposures (e.g. fisting) in our study. However analysis of sexual risk was not the primary aim of the study and is an acknowledged weakness although other investigators have reported that unprotected traumatic anal sex, bleeding during sex, fingering, fisting, rimming, and number of recent sexual partners are risks for possible sexual transmission [6,7,29].

Under-reporting of IDU may have also biased our study. Previous studies in Australia have shown however the social desirability bias (e.g. under reporting of IDU) is uncommon in Australia compared to the US or UK [30,31]. Furthermore those who tested positive for HCV had generally been attending the centre's service and seeing the same clinician for years (mean attendance 5 years) and were therefore likely to have established good rapport with their clinicians.

## Conclusions

A considerable proportion of HIV positive MSM who did not use intravenous drugs contracted HCV, presumably via sexual transmission and the majority was investigated for HCV because of abnormal liver enzymes.

## Abbreviations

HCV: Hepatitis-C virus; HIV: Human Immunodeficiency Virus; MSM: Men having Sex with Men; IDU: injecting drug use

## Competing interests

The authors declare that they have no competing interests.

## Authors' contributions

All authors contributed to conception, design and interpretation of data. DGG, TRHR and CKF drafted and reviewed the manuscript. JSH assisted in statistical analysis. All authors reviewed and approved the final manuscript.

## Pre-publication history

The pre-publication history for this paper can be accessed here:

http://www.biomedcentral.com/1471-2334/11/39/prepub

## References

[B1] Joseph J Stoff DM van der Horst C HIV/hepatitis C virus co-infection: basic, behavioral and clinical research in mental health and drug abuse AIDS 2005 19 suppl 3 S3 S7 10.1097/01.aids.0000192063.01658.48 16251825

[B2] David L Thomas MD HIV/HCV co infection: comorbidity and clinical implications Adv Stud Med 2005 5 4C S352 5

[B3] National Centre in HIV Epidemiology and Clinical Research (NCHECR) (2009) Annual Surveillance Report HIV/AIDS, viral hepatitis and sexually transmissible infections in Australia[online] 2009 http://www.nchecr.unsw.edu.au/NCHECRweb.nsf/resources/SurvReports_3/$file/ASR2009-updated-2.pdf cited 29/06/2010 10.33321/cdi.2004.28.31 15460970

[B4] Salmon-Ceron D Lewden C Morlat P Bevilacqua S Jougla E Bonnet F Liver disease as a major cause of death among HIV infected patients: role of hepatitis C and B viruses and alcohol J Hepatol 2005 42 6 799 805 10.1016/j.jhep.2005.01.022 15973779

[B5] Alter MJ Prevention of spread of hepatitis C Hepatology 2002 36 5 Suppl 1 S93 8 10.1002/hep.1840360712 12407581

[B6] Turner JM Rider AT Imrie J Copas AJ Edwards SG Dodds JP Behavioural predictors of subsequent hepatitis C diagnosis in a UK clinic sample of HIV positive men who have sex with men Sex Transm Infect 2006 82 298 300 10.1136/sti.2005.018366 16877578 PMC2564713

[B7] Rauch A Rickenbach M Weber R Hirschel B Tarr PE Bucher HC Unsafe sex and increased incidence of hepatitis C virus infection among HIV-infected men who have sex with men: the Swiss HIV Cohort study Clin Infect Dis 2005 41 395 402 10.1086/431486 16007539

[B8] Ghosn J Deveau C Goujard C Garrigue I Saichi N Galimand J Increase in hepatitis C virus incidence in HIV-1-infected patients followed up since primary infection Sex Transm Infect 2006 82 458 460 10.1136/sti.2006.021493 16923739 PMC2563871

[B9] van de Laar TJW Van der Bij AK Prins M Bruisten SM Brinkman K Ruys TA Increase in HCV incidence among men who have sex with men in Amsterdam most likely caused by sexual transmission J Infect Dis 2007 196 230 8 10.1086/518796 17570110

[B10] Jin F Prestage GP Matthews G Zablotska I Rawstorne P Kippax SC Prevalence, Incidence and risk factors for hepatitis C in homosexual men: data from two cohorts of HIV-positive men in Sydney, Australia Sex Trans Infect 2010 86 25 8 10.1136/sti.2009.038182 19841001

[B11] Bodsworth NJ Cunningham P Kaldor J Donovan B Hepatitis C virus infection in a large cohort of homosexually active men: independent associations with HIV-1 infection and injecting drug use but not sexual behaviour Genitourin Med 1996 72 118 22 8698359 10.1136/sti.72.2.118 PMC1195621

[B12] Donahue JG Nelson KE Munoz A Vlahov D Rennie LL Taylor EL Antibody to hepatitis C virus among cardiac surgery patients, homosexual men, and intravenous drug users in Baltimore, Maryland Am J Epidemiol 1991 134 10 1206 11 1720924 10.1093/oxfordjournals.aje.a116023

[B13] van de Laar TJW Matthews GV Prins M Danta M Acute hepatitis C in HIV infected men who have sex with men: an emerging sexually transmitted infection AIDS 2010 24 1799 1812 10.1097/QAD.0b013e32833c11a5 20601854

[B14] National Health and Medical research council A strategy for detection and management of HCV in Australia, Commonwealth department of Health and Family health services, Commonwealth of Australia 1997 http://www.nhmrc.gov.au/_files_nhmrc/file/publications/synopses/withdrawn/cd14.pdf cited 19/07/2010

[B15] Thomson E Nastouli E Main J Karayiannis P Eliahoo J Muir D McClure MO Delayed anti-HCV antibody response in HIV-positive men acutely infected with HCV AIDS 2009 23 89 93 10.1097/QAD.0b013e32831940a3 19050390 PMC2646374

[B16] Fairley CK Hoy J Leslie DE Nicholson S Gust ID The development of hepatitis C antibody shortly after acute icteric non A non B hepatitis Med J Aust 1992 156 6 387 9 1372078 10.5694/j.1326-5377.1992.tb139840.x

[B17] STIGMA STI testing guideline Sexually Transmitted infection testing guidelines for Men who have sex with men 2008 2010 http://www.ashm.org.au/images/publications/guidelines/stigma_sti_testing_guidelines_for_msm.pdf

[B18] Richardson D Fisher M Sabin CA Sexual transmission of hepatitis C in MSM may not be confined to those with HIV infection J Infect Dis 2008 197 8 1213 4 10.1086/533454 18462168

[B19] Alary M Joly JR Vincelette J Lavoie R Turmel B Remis RS Lack of evidence of sexual transmission of hepatitis C virus in a prospective cohort study of men who have sex with men Am J Public Health 2005 95 502 505 10.2105/AJPH.2003.020388 15727984 PMC1449209

[B20] van der Helm J Geskus RB del Amo J Cheˆne G Gill J Hamouda O Hepatitis C epidemic among HIVR men who have sex with men started before 2000 [abstract 643] 17th Conference on Retroviruses and Opportunistic Infections(CROI) 2010 San Francisco, USA

[B21] Stellbrink HJ Schewe CK Vogel M Hoffmann C Noah C Incidence, genotype distribution, and prognosis of sexually transmitted acute hepatitis C in a cohort of HIV-infected patients [abstract 645] 17th Conference on Retroviruses and Opportunistic Infections (CROI) 2010 San Francisco, USA

[B22] Melbye M Biggar RJ Wantzin P Krogsgaard K Ebbesen P Becker NG Sexual transmission of hepatitis C virus: cohort study (1981-9) among European homosexual men BMJ 1990 301 210 212 10.1136/bmj.301.6745.210 2118402 PMC1663579

[B23] Giuliani M Caprilli F Gentili G Maini A Lepri AC Prignano G Incidence and determinants of hepatitis C virus infection among individuals at risk of sexually transmitted diseases attending a human immunodeficiency virus type 1 testing program Sex Transm Dis 1997 24 533 537 10.1097/00007435-199710000-00007 9339972

[B24] Browne R Asboe D Gilleece Y Alkins M Mandalia S Gazzard B Increase number of acute hepatitis C infection in HIV positive homosexual men; is sexual transmission feeding the increase? Sex Transm Infect 2004 80 326 7 10.1136/sti.2003.008532 15295139 PMC1744861

[B25] Thomson E Nastouli E Main J Karayiannis P Eliahoo J Muir D McClure MO Delayed anti-HCV antibody response in HIV-positive men acutely infected with HCV AIDS 2009 23 89 93 10.1097/QAD.0b013e32831940a3 19050390 PMC2646374

[B26] Lee D Chen MY Fairley CK The re-emergence of syphilis among homosexually active men in Melbourne Aust N Z J Public Health 2005 29 390 1 10.1111/j.1467-842X.2005.tb00215.x 16222942

[B27] Bissessor M Fairley CK Leslie D Howley K Chen MY Frequent Screening for Syphilis as Part of HIV Monitoring Increases the Detection of Early Asymptomatic Syphilis Among HIV-Positive Homosexual Men J Acquir Immune Defic Syndr 2010 55 211 216 10.1097/QAI.0b013e3181e583bf 20585261

[B28] Morton AN Fairley CK Zaia AM Chen MY Anorectal lymphogranuloma venereum in a Melbourne man Sexual Health 2006 3 189 190 10.1071/SH06029 17044226

[B29] Denta M Brown D Bhagani S Pybus OG Sabin CA Nelson M Recent epidemic of acute hepatitis C virus in HIV infected men who have sex with men linked to high-risk sexual behaviours AIDS 2007 21 983 91 10.1097/QAD.0b013e3281053a0c 17457092

[B30] Tideman RL Chen MY Pitts MK Ginige S Slaney M Fairley CK A randomized trial comparing computer-assisted with face-to-face sexual history taking in a clinical setting Sex Transm Inf 2007 83 52 6 10.1136/sti.2006.020776 PMC2598599 17098771

[B31] Fairley CK Sze JK Chen MY Computer assisted self interviewing in sexual health clinics Sex Trans Dis 2010 37 11 665 668 10.1097/OLQ.0b013e3181f7d505 20975481

